# Bridging the gap: integrating plant physiology and soil science in nanotechnology and biochar research for sustainable agriculture

**DOI:** 10.3389/fpls.2025.1661442

**Published:** 2025-08-18

**Authors:** Luana Vanessa Peretti Minello, Kettlin Ruffatto, Fernanda Maria Corrêa, Leonardo Fluck Mariani, Iftikhar Ahmad, Raul Antonio Sperotto

**Affiliations:** ^1^ Botany Department, Biology Institute, Graduate Program in Plant Physiology, Federal University of Pelotas, Pelotas, Brazil; ^2^ Graduate Program in Biotechnology, University of Vale do Taquari - Univates, Lajeado, Brazil

**Keywords:** biochar, nanoparticles, plant physiology, soil science, sustainable agriculture

## 1 Introduction

**Figure 1:**
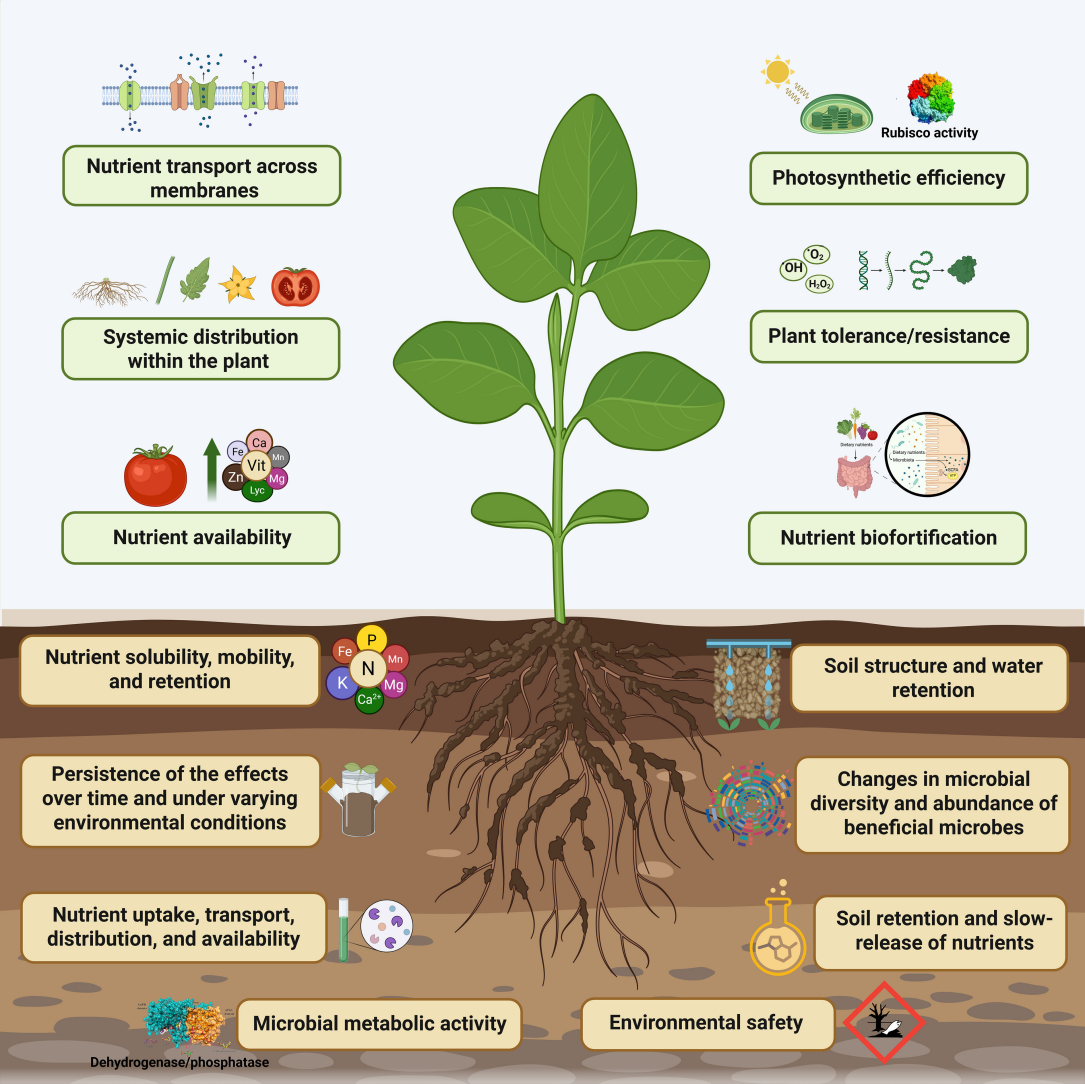
Essential physiological and soil-related assessments that researchers should incorporate to improve the scientific rigor, practical relevance, and agricultural applicability of their results.

The application of nanotechnology and biochar in agricultural systems has gained significant attention in recent years due to their potential to enhance nutrient availability, improve plant stress tolerance/resistance, increase plant productivity, and promote sustainable farming practices (Bamdad et al., 2022; Hasnain et al., 2023; Ahmed et al., 2024; Gill et al., 2024; Manzoor et al., 2024; Rana et al., 2024; Verma et al., 2024; Yasin et al., 2024). However, there is growing concern within the scientific community about the frequent lack of integration between fundamental plant physiology and soil science in studies involving these materials (Chadha et al., 2024; Maaz et al., 2025). As highlighted by Frank and Husted (2024) and Husted et al. (2024), numerous publications in this field suffer from flawed experimental designs, unrealistic application regimes, and superficial data interpretation, often leading to conclusions that lack depth and are difficult to translate into practical and sustainable agronomic solutions. Other studies focus primarily on material characterization or yield improvements without a comprehensive understanding of how these amendments interact with plant physiological processes and soil dynamics (Husted et al., 2024; Shani et al., 2024). To bridge this gap, we argue that future research must prioritize a systematic and detailed understanding of how nanomaterials and biochar influence plant nutrient uptake, stress responses, and photosynthetic efficiency, alongside their impacts on soil physicochemical properties and microbial interactions. In this Opinion article, we highlight key physiological and soil science analyses that researchers should consider to enhance the robustness, relevance, and agronomic applicability of their findings (**Figure 1**).

## 2 Key physiological considerations

### 2.1 Nutrient uptake, assimilation and biofortification

One of the primary motivations for applying nanomaterials and biochar in agriculture is their potential to enhance nutrient availability and uptake. In some specific cases, nanofertilizers have been shown to improve the bioavailability and efficiency of essential nutrients like nitrogen, phosphorus, and potassium, leading to better nutrient uptake by plants and reduced environmental losses (Alam et al., 2024; Arora et al., 2024; Chadha et al., 2024; Saurabh et al., 2024; Zhang et al., 2024). Similarly, biochar applications have been found to improve soil nutrient retention and availability, thereby enhancing nutrient uptake and crop productivity (Hossain et al., 2020; Bekchanova et al., 2024; Ullah et al., 2024; Upadhyay et al., 2024). However, many studies fail to assess the fundamental processes by which these materials influence root absorption, nutrient transport across membranes, and systemic distribution within the plant.

Root absorption and *in planta* translocation of nutrients and nanomaterials can be investigated using a range of complementary analytical techniques. For example, laser ablation inductively coupled plasma mass spectrometry (LA-ICP-MS) allows high-resolution spatial mapping of isotopes within plant tissues, providing detailed insights into elemental distribution (Cui et al., 2023). Confocal Raman microscopy offers a non-destructive imaging approach to visualize particles and chemical compounds in cells and tissues (Saletnik et al., 2021), while transmission electron microscopy (TEM) can confirm the presence and localization of nanoparticles at the cellular and subcellular levels (Ďúranová et al., 2024). Radioactive tracers combined with ICP-MS analyses provide quantitative data on nutrient uptake and translocation over time (Di Tullo et al., 2015).

For understanding elemental speciation, oxidation states, and coordination environments, synchrotron-based techniques such as micro-X-ray fluorescence (μ-XRF) and X-ray absorption spectroscopy (XAS) are particularly powerful. μ-XRF enables high-resolution elemental mapping, whereas XAS provides detailed chemical information including oxidation state, interatomic distances, and elemental speciation (Vijayan et al., 2015). When combined, these synchrotron methods deliver a comprehensive picture of spatial distribution and chemical form within plant tissues (Zhao et al., 2022). However, despite their strengths, synchrotron techniques have limited accessibility due to the need for specialized facilities and beamtime allocation (Ashe et al., 2025). Furthermore, we recognize that in developing countries or institutions with limited budgets, access to these techniques is often restricted due to high costs and a lack of training and expertise in data acquisition and interpretation. Therefore, we strongly advocate for more collaborative scientific efforts worldwide to help overcome these obstacles.

Complementing these imaging and spectroscopic approaches, transcriptomic and proteomic analyses can elucidate molecular responses by revealing changes in transporter gene and protein expression, thereby providing mechanistic insights into nutrient uptake and translocation pathways (Mostofa et al., 2022). Although combining these techniques can make data analysis and interpretation more complex and require careful consideration, they can provide a more comprehensive understanding of plant physiological responses to the types of nanomaterials applied (Pinheiro et al., 2024).

Beyond improving general nutrient uptake, nanotechnology and biochar hold promise for biofortifying edible plants with essential microutrients. Several studies suggest that nanoparticles loaded with these micronutrients can enhance their bioavailability in soils and increase their accumulation in edible plant tissues (Shafiq et al., 2023; Ahmad et al., 2024; Huang et al., 2024). Likewise, biochar has been reported to improve soil retention and slow-release properties for key micronutrients, potentially boosting their uptake and accumulation in edible parts (Awad et al., 2017; Ahmed et al., 2024; Bañuelos et al., 2025). Despite promising increases in micronutrient content, rigorous absorption and utilization studies are often lacking, making it difficult to confirm that these technologies truly enhance micronutrient bioavailability in ways that improve human nutritional outcomes (Bechoff and Dhuique-Mayer, 2017; Altemimi et al., 2024). Future research should incorporate biofortification-specific methodologies, including nutrient speciation analysis using XAS technique (described above), bioavailability studies using simulated *in vitro* gastric and intestinal digestion assays (Hu et al., 2019), and human dietary impact assessments (Jackson et al., 2024). Additionally, long-term field trials and multi-environmental evaluations are essential to determine the consistency of these approaches in real agricultural settings and their potential impact on human nutrition.

### 2.2 Stress response mechanisms

Nanomaterials and biochar have been proposed as tools to improve plant tolerance to abiotic stresses such as drought (Rajhi et al., 2024; Shirvani-Naghani et al., 2024), salinity (Gao et al., 2024; Soni et al., 2024), and heavy metal toxicity (Ghorbani et al., 2024; Shahzad et al., 2024). While many studies report improvements in biomass and yield following nanomaterial or biochar applications under abiotic stress, there is a notable lack of deeper physiological and cellular assessments. A few studies, such as Waseem et al. (2023), which emphasizes the need for integrated morphological, physiological, biochemical, and molecular metrics, and Wang et al. (2023), which combines biomass data with photosynthetic performance and isotopic indicators, demonstrate the type of mechanistic insight needed. Measuring oxidative stress markers (e.g., reactive oxygen species, antioxidant enzyme activity, membrane stability index, proline accumulation, and lipid peroxidation/protein carbonylation levels) can provide deeper insights into the protective or detrimental effects of these materials (Khan et al., 2024; Zeeshan et al., 2024). Additionally, hormonal profiling should be performed to determine whether they influence phytohormones such as abscisic acid (ABA), jasmonic acid (JA), and salicylic acid (SA), which play crucial roles in stress signaling (Adhikari et al., 2023; Wang et al., 2024). Integrating omics approaches, including transcriptomics, proteomics, and metabolomics, into these studies is essential to uncover the molecular mechanisms underlying the beneficial effects of nanoparticles and biochar, ultimately improving their targeted application in stress mitigation strategies.

Beyond abiotic stress tolerance, nanotechnology and biochar have been explored for their potential to enhance plant resistance to biotic stresses, including pathogen infections and herbivore attacks (Waqas et al., 2018; Ruffatto et al., 2025). Some nanoparticles have demonstrated antimicrobial properties, reducing disease incidence in various crops (Islam et al., 2024; Ogunyemi et al., 2024), while others have been shown to activate plant defense mechanisms against herbivory, exhibiting strong insecticidal effect (Hemalatha et al., 2024; Mawale and Giridhar, 2024). Biochar produced from deciduous trees, dolomite, and molasses has been reported to enhance plant resistance to herbivory by increasing JA levels (Waqas et al., 2018). In another study, bamboo biochar improved plant resistance to fungal infections by activating stress signaling pathways and strengthening the immune system (Zhu et al., 2021). However, many studies in this field fail to comprehensively assess plant immune responses at the molecular level, highlighting the need for deeper investigations into the underlying biochemical and genetic mechanisms, as also emphasized by Singh et al. (2024), who underscore that the detailed mechanisms of nanomaterial–plant interactions remain underexplored. Future research should incorporate transcriptomic and metabolomic approaches to assess changes in plant defense gene expression and secondary metabolite production. Additionally, studies should investigate whether biochar and nanoparticles can prime plants for induced resistance, a mechanism by which plants develop a heightened state of defense against subsequent pathogen attacks.

### 2.3 Photosynthetic performance and water use efficiency

Improvements in photosynthetic efficiency and water use efficiency (WUE) are frequently claimed as benefits of nanomaterials and biochar application (Chattha et al., 2022; Wu et al., 2024). However, photosynthesis is often evaluated solely through chlorophyll content or net CO_2_ assimilation rate, missing critical underlying processes. Detailed gas exchange measurements should be coupled with chlorophyll fluorescence analysis (e.g., Fv/Fm, NPQ) to dissect photochemical efficiency and non-photochemical energy dissipation under stress conditions, as demonstrated in *Camellia sinensis* tea cultivation studies (Chen et al., 2024). Moreover, stomatal behavior, mesophyll conductance, and the activity of key enzymes such as Rubisco should be investigated to determine whether observed improvements are due to intrinsic physiological changes rather than indirect effects (e.g., improved soil water retention) (Sheng-Lan et al., 2022). Furthermore, long-term studies under field conditions are essential to determine whether these observed improvements in photosynthetic efficiency and WUE translate into sustained benefits for plant growth, yield, and resilience, considering that different environmental conditions can significantly influence photosynthetic responses and overall plant performance.

## 3 Soil science considerations

### 3.1 Bioavailability and soil-nutrient interactions

Nanoparticles and biochar can modify nutrient bioavailability in complex ways (Jafari and McClements, 2017; Li and Li, 2022; Rana et al., 2024), but many studies measure only total nutrient content in soil and plants without considering bioavailable fractions. It is already known that failing to account for different chemical forms of nutrients can lead to misleading conclusions, as total concentration does not necessarily reflect what is accessible for plant uptake (Rahman and Schoenau, 2022). The interactions between nanoparticles, biochar, and soil components can influence nutrient solubility, mobility, and retention, affecting how efficiently plants can absorb and utilize these elements (Forján et al., 2024). For instance, nanoparticles may enhance nutrient bioavailability by preventing fixation in the soil matrix or acting as nutrient carriers, using controlled-release formulations and biopolymeric encapsulation approaches that limit nutrient loss and facilitate targeted root uptake (Beig et al., 2022; Dutta et al., 2022; Rana et al., 2024). Similarly, biochar can act as a reservoir, gradually releasing nutrients over time (Chanda et al., 2025). However, the extent of these effects depends on factors such as soil pH, organic matter content, and the physicochemical properties of the applied materials (Forján et al., 2024).

To accurately assess these impacts, sequential extraction methods (Filgueiras et al., 2002) should be employed to distinguish between readily available, exchangeable, and strongly bound nutrients. These techniques provide a clearer picture of nutrient dynamics, helping to determine whether observed increases in total nutrient content translate into real agronomic benefits. Additionally, speciation analysis using the XAS technique can determine how elements change oxidation state and binding forms in the presence of these materials (Dong et al., 2022). This is particularly relevant for micronutrients that exist in multiple oxidation states, as their bioavailability is directly influenced by their chemical speciation. Moreover, long-term studies incorporating soil incubation experiments and plant uptake trials should be conducted to evaluate the persistence of these effects over time and under varying environmental conditions (Rajput et al., 2024). By integrating these advanced analytical approaches, future research can move beyond simplistic assessments and develop a more functional understanding of how nanoparticles and biochar influence nutrient cycling in agricultural systems.

### 3.2 Microbial community dynamics

The effects of nanomaterials and biochar on soil microbial communities are often overlooked, despite their critical role in nutrient cycling and plant health (Bamdad et al., 2022; Bolan et al., 2024; Cao et al., 2024). A better understanding of these interactions is essential, as the lack of microbial data makes it difficult to predict the long-term impacts on soil health. Therefore, further studies should employ high-throughput sequencing and metagenomic approaches to assess changes in microbial diversity, the functional expression of genes related to soil nitrogen mineralization, nitrate reduction to ammonium, and soil nitrogen assimilation, as well as the abundance of beneficial microbes such as mycorrhizal fungi and nitrogen-fixing bacteria (Ansari et al., 2024; Zhu et al., 2024; Reid et al., 2025). Moreover, enzyme activity assays (e.g., dehydrogenase, phosphatase) can provide additional insights into microbial metabolic activity in treated soils (Das et al., 2025). Understanding these microbial shifts will be crucial for optimizing nanomaterial and biochar applications in agriculture, ensuring they promote beneficial microbial interactions while minimizing potential disruptions to soil ecosystems.

Furthermore, multiomics techniques can aid in understanding complex processes related to plant-microbiota interactions, such as nitrogen fixation, induction of systemic resistance, and mycorrhizal association (Jain et al., 2024). Studies integrating multiomics and bioinformatics techniques have revealed that the diversity of microbiota present in soil, even when contaminated, can increase the content of antioxidants and phytohormones in plants, ensuring the trade-off between defense and production (Sengupta and Pal, 2021). Furthermore, understanding the metabolites released during plant-microorganism interactions may offer promising insights for the development of next-generation inoculants capable of improving plant growth and development (Mishra et al., 2022).

### 3.3 Soil physicochemical properties and environmental safety

Changes in soil properties induced by nanomaterials and biochar, such as water-holding capacity, porosity, cation exchange capacity (CEC), and aggregate stability, significantly influence plant responses (Nepal et al., 2023; Kumar et al., 2025). However, these properties are often insufficiently characterized. Standard soil science methodologies, including BET surface area analysis for porosity (Blattmann and Plötze, 2024), laser diffraction for particle size distribution (Gresina et al., 2025), and rheological measurements for soil consistency (Javaheri et al., 2021), should be integrated into future research. Incorporating these methodologies will provide a more comprehensive understanding of how nanomaterials and biochar alter soil structure and function, ultimately improving their application for sustainable soil management and crop productivity.

Additionally, the environmental safety of nanomaterials and biochar applications in soils remains an underexplored aspect. While biochar is generally considered environmentally friendly (Chueangchayaphan et al., 2025), the use of unsuitable biomass feedstocks, suboptimal preparation conditions, or inappropriate production methods can lead to the formation of harmful compounds (Xiang et al., 2021). Some engineered nanomaterials may accumulate in soils, posing potential risks to microbial communities, water quality, and non-target organisms (Arora et al., 2022). Future studies should assess the long-term persistence, mobility, and potential toxicity of these materials, ensuring that their application does not lead to unintended ecological consequences (Godlewska et al., 2021; Tran et al., 2024). A holistic risk assessment framework integrating ecotoxicological studies, soil health indicators, and regulatory guidelines will be essential to ensure the safe and sustainable use of nanomaterials and biochar in agricultural systems (Iavicoli et al., 2017; de Oliveira Pereira et al., 2020).

## 4 A call for holistic experimental designs

A significant limitation in current research is the predominant focus on evaluating nanomaterials and biochar under controlled conditions, often overlooking agronomic variability. While greenhouse and laboratory studies provide valuable insights, field trials are essential for validating these findings under real-world conditions. However, these trials should extend beyond yield measurements to include comprehensive physiological and soil analyses, allowing for a deeper understanding of the mechanisms driving plant responses. Furthermore, a critical yet often overlooked aspect is the rigorous physicochemical characterization of the materials used, including particle size, charge, surface structure, dissolution behavior, and composition, as these properties strongly influence their behavior and efficacy in agricultural environments. Adopting a multidisciplinary approach, integrating expertise from plant physiology, soil science, agronomy, and material science, will ensure that research generates biologically relevant and agronomically applicable data.

As the application of nanotechnology and biochar in agriculture continues to grow, research must move beyond surface-level evaluations of plant growth and yield. Incorporating physiological and soil-based assessments will strengthen the scientific foundation of these studies and enhance their practical relevance. A focus on underlying processes and biological mechanisms, coupled with well-designed experiments, will ensure that nanomaterials and biochar contribute effectively to the development of resilient and productive agricultural systems, bridging the gap between experimental findings and field-scale implementation. Furthermore, the development of additional public policies supporting research focused on the application of nanotechnology and biochar in agriculture is necessary.
